# The effects of exercise variation in muscle thickness, maximal strength and motivation in resistance trained men

**DOI:** 10.1371/journal.pone.0226989

**Published:** 2019-12-27

**Authors:** Eneko Baz-Valle, Brad J. Schoenfeld, Jon Torres-Unda, Jordan Santos-Concejero, Carlos Balsalobre-Fernández

**Affiliations:** 1 Department of Physical Education and Sport, University of the Basque Country UPV/EHU, Vitoria-Gasteiz, Spain; 2 Health Sciences Department, Bronx, NY, United States of America; 3 Department of Physiology, Faculty of Medicine and Nursing, University of the Basque Country UPV/EHU, Vitoria-Gasteiz, Spain; 4 Department of Physical Education, Sport and Human Movement, Autonomous Univerisity of Madrid, Madrid, Spain; Universidade Federal de Mato Grosso do Sul, BRAZIL

## Abstract

**Background:**

The objective of the present study was to compare the effects of a traditional resistance training program (fixed exercises and repetition ranges) to a resistance training program where exercises and repetition ranges were randomized on a session-by-session basis on markers of muscular adaptations and intrinsic motivation.

**Methods:**

Twenty-one resistance trained men were randomized to perform an 8-week resistance training program using either a fixed exercise selection (CON) or having exercises randomly varied each session via a computerized app. Both groups performed 3 sets of 6 exercises, with training carried out 4 times per week.

**Results:**

Both conditions promoted large, statistically significant increases in the bench press and back-squat 1 repetition maximum without differences between groups. Muscle thickness (MT) measures for the individual quadriceps showed large, statistically significant increases in of the vastus lateralis and rectus femoris for both conditions, with no observed between-group differences. Although no between-group in MT were noted for the vastus intermedius, only the CON displayed significant increases from baseline. Participants in EXP showed a significant, moderate improvement in the intrinsic motivation to training, while participants in the CON group presented non-significant decreases in this variable.

**Conclusions:**

Varying exercise selection had a positive effect on enhancing motivation to train in resistance-trained men, while eliciting similar improvements in muscular adaptations.

## Introduction

Resistance training (RT) is well-established as an effective method to increase muscle mass, strength and overall health in different populations [1–5]. It has been proposed that proper manipulation of RT variables may help to optimize muscular adaptations [2,6]. Practitioners can manipulate a variety of RT variables to elicit desired muscular adaptations. These include both quantitative variables, such as training volume, frequency, rest intervals or cadence [1,6,7], and qualitative variables, such as exercise selection. For example, with respect to training load, it has been shown that, when volume is equated, both light (i.e., <50% 1-RM) and heavy (i.e., > 80%1-RM) loads can elicit similar hypertrophic responses [8], while heavy loading seems to elicit greater increases in maximal strength [9].

Gaining muscle mass and strength while maintaining or increasing motivation to exercise seem to be a relevant factor to improve adherence to exercise. In this sense, some popular exercise programs advocate frequent rotation of exercises as a means to optimize results and improve exercise motivation [10]. The term “muscle confusion” has been coined to describe the effects of constantly varying exercise selection as a means to provide a novel stimulus that enhances muscular adaptations [10]. However, research on the topic is limited. Fonseca et al. [11] showed that changing lower body exercises every two weeks may elicit greater regional-specific hypertrophy of the quadriceps muscle compared to just performing the squat. More recently, Rauch et al. [12], demonstrated that varying exercise selection via autoregulation produced modestly greater increases in lean mass and strength compared to a fixed exercise protocol. However, to our knowledge, no study to date has endeavored to investigate the effects of randomly undulating exercise selection as some programs advocate. It is conceivable that such frequent rotation of exercises may enhance results by continually providing a novel stimulus to muscles and/or bolstering motivation to train.

The objective of the present study was to compare the effects of a traditional training program (fixed exercises and repetition ranges) to a training program where exercises and repetition ranges were randomized on a session-by-session basis on markers of muscular adaptations and intrinsic motivation in resistance trained men. We hypothesized that the random routine would increase intrinsic motivation without hampering gains in muscle mass and strength.

## Material & methods

### Participants

Twenty-one healthy men (age = 23.4 ± 3.5 years; body-mass = 77.5 ± 11 kg; body-height = 1.78 ± 0.05 m; body-fat = 13.6 ± 2.5%; lean body mass = 86.3 ± 2.5%) with at least 2 years of experience with resistance training voluntarily joined this investigation. Participants were required to meet the following inclusion criteria: 1) men between the ages of 18–35; 2) no existing musculoskeletal disorders; 3) claimed to be free from consumption of anabolic steroids or any other illegal agents known to increase muscle size; 4) experienced with RT, defined as consistently lifting weights at least 3 times per week for a minimum of 2 years. A total of 19 participants completed the study; two participants dropped out prior to completion, for personal reasons. Written informed consent was obtained from each participant after a thorough explanation of the testing protocol, the possible risks involved, and the right to terminate participation at will. The study was approved by the Institutional Review Board of the University of the Basque Country, Spain (ref. 2018/099) and all procedures were in accordance with the declaration of Helsinki (2013).

### Training interventions

Participants were randomly allocated to either an experimental group (EXP) or a control group (CON). Participants in the CON group carried out an 8-week resistance training program consisting of 3 sets of 6 exercises performed 4 times per week. On Monday and Thursday, participants performed an upper-body workout, while on Tuesday and Friday they performed a lower-body workout, for a total of 32 RT sessions. Upper body exercises in CON group included bench-press, pendlay row, shoulder press, latpull down, dumbbell fly and dumbbell pull-over, while the lower body exercises included back squat, deadlift, leg press, hip thrust, leg extension and leg curl. Training load was linearly periodized by reducing the number of repetitions per set every 2 weeks, from 12RM to 6RM. Thus, there were a total of 8 training sessions with each XRM. See Table 1 for more details.

**Table 1:** Distribution of training load (number of repetitions to failure per) set through the intervention in both the control and experimental groups.

	Week 1	Week 2	Week 3	Week 4	Week 5	Week 6	Week 7	Week 8
Control group*	12 RM	12 RM	10 RM	10 RM	8 RM	8 RM	6 RM	6 RM
*Experimental group*								
Day 1 (upper-body)	8 RM	6 RM	6 RM	10 RM	12 RM	12 RM	10 RM	12 RM
Day 2 (lower-body)	10 RM	12 RM	12 RM	10 RM	6 RM	8 RM	8 RM	10 RM
Day 3 (upper-body)	10 RM	8 RM	12 RM	6 RM	10 RM	8 RM	8 RM	6 RM
Day 4 (lower-body)	8 RM	6 RM	6 RM	12 RM	8 RM	12 RM	10 RM	6 RM

*Participants in the control group performed the same number of repetitions to failure per set each day of the week

Participants in EXP group carried out a resistance training program with the same duration and sessions per week as CON, but with exercises randomly chosen each session from a computerized database of 80 different exercises via an iPhone app (Ace Workout) specifically designed for the present study. The randomization algorithm was written to select 3 pulling (e.g., pull-up, lat-pull down and pull-over) and 3 pushing (e.g., bench-press, standing military press and dumbbell flies) exercises for the upper-body, with no exercise repeated within the same workout. For the lower-body, the algorithm chose 3 exercises with greater participation of the anterior chain (ex., back-squat, leg extension and leg press) and 3 for the posterior chain (e.g., deadlift, hip-thrust and leg curl). Both EXP and CON were afforded two minutes rest between sets. Total training volume (measured as total number of sets and repetitions) was equated between groups (see Table 1 for more details). All participants took part in at least 95% of the training sessions.

### Psychological measures

One day before and one day after the training intervention, the intrinsic motivation and demotivation factors of the Situational Motivation Scale were measured using a validated Spanish version of this questionnaire [13]. A total of 15 out of 19 participants completed the questionnaire online using an ad-hoc form that was sent to them, while 4 participants did not complete it for unspecified reasons. The validity of the intrinsic motivation factor was confirmed using Cronbach’s alpha (α > 0.8).

### Muscle thickness

Muscle thickness (MT) was measured using B-mode ultrasound imaging (GE LOGIQTM e, GE Healthcare, WI, USA) with a linear-array transducer (code 12L-RS, variable frequency band 4.2–13.0 Mhz). Measurements were performed with participants supine, with arms and legs extended and relaxed. Prior to testing, participants remained in this position for 10 minutes to allow for stabilization of normal body fluids. The technician then applied a water-soluble transmission gel (Aquasonic 100 Ultrasound Transmission gel; Parker Laboratories Inc., Fairfield, NJ, USA) to each measurement site and a 5 MHz ultrasound probe was placed perpendicular to the tissue interface without depressing the skin. When the quality of the image was deemed as satisfactory, the technician saved the image to the hard drive and obtained MT dimensions of the *vastus lateralis* (VL) and *rectus femoris* (RF) by measuring the distance from the subcutaneous adipose tissue-muscle interface to the muscle-bone interface as detailed in previous research [14,15]. Measurements for the *vastus intermedius* (VI) were obtained at the widest distance between the bony surface of the femur and RF muscle interface [14]. Distances were measured using the straight-line function of ImageJ software. Measurements were taken on the right side of the body at two different sites: *medial quadriceps femoris*, and *lateral quadriceps femoris*. For the *quadriceps femoris*, measurements were taken 50% between the lateral condyle of the femur and greater trochanter for the medial (RF and VI) and lateral (VL) aspects of the thigh. Three images were taken at each site and the values were averaged to obtain a final measurement. In an effort to help ensure that swelling in the muscles from training did not obscure results, images were obtained 48–72 h before commencement of the study and after the final training session. This timeframe is consistent with research showing that acute increases in MT return to baseline within 48 h following a resistance training session [16].

### Body composition

One day before starting and one day after ending the 8-week intervention, body composition was measured using anthropometric methods. Participants were weighed on a calibrated digital scale whilst wearing minimal clothing. Height was measured with a stadiometer attached to the scale with participants standing shoeless and head aligned in the horizontal Frankfurt plane. Body Mass Index (BMI) was calculated as follows: total body mass (in kg) stature (in m) -2. Seven-site skinfold measurements (in mm) were taken from the biceps, triceps, scapular, abdominal, suprailiac, thigh and medial calf sites according to standard procedures using a skin fold caliper (Harpenden1, Baty International, West Sussex, UK). All skinfolds were measured to the nearest 1 mm and the mean of 3 readings was recorded as the final value at each site. All body composition measurements were taken by the same investigator 24-48h before and 24-48h after completion of the training protocol. Body fat percentage was estimated using the equation proposed by Faulkner [17].

### Maximal dynamic strength

Subjects reported to the laboratory having refrained from any exercise other than activities of daily living for at least 48 hours prior to baseline testing and at least 48 hours prior to testing at the conclusion of the study. Maximal dynamic strength on the free-weight barbell bench-press and back squat exercises were measured before and after the training intervention via the 1-repetition maximum (1-RM) test [6]. All participants were familiar with 1-RM testing, and prior to testing were asked for their previous 1-RM value for each exercise. They subsequently performed 2 repetitions at 60, 70 and 80% and 1 repetition at 90 and 100% 1-RM. If a participant failed an attempt, the load was reduced in a range of 2.5-5kg to determine their 1-RM with a high degree of precision. Three minutes of passive rest were afforded between each trial.

### Dietary adherence

To avoid the potential for dietary confounding, subjects were instructed to maintain their usual and customary eating habits while consuming a minimum protein intake of 2g/kg and a eucaloric diet or slight energy surplus. To assess nutritional adherence, subjects tracked their meals with a nutritional tracking app (http://www.myfitnesspal.com) at the beginning and end of the intervention, providing data related to total consumed energy, as well as proteins, fat and carbohydrate distribution. Data were tracked at the beginning and the end of the study to ensure dietary adherence. Subjects agreed not to consume any supplement that could interfere with the studied outcomes (such as creatine and whey protein) throughout the investigation period.

### Statistical analyses

We tested all variables for normal distribution (Shapiro-Wilk test) and homogeneity of variances (Levene’s test). Data are presented as mean with standard deviations. An independent samples T-test was carried out on pre-intervention muscle thickness data to check for potential differences between groups. An Analysis of Covariance (ANCOVA) was employed to determine the potential differences between groups on the post-intervention scores, with the pre-intervention scores used as a covariate. Cohen’s *d* effect size (ES) with 95% CIs were calculated to analyze the magnitude of the potential pre-post intervention differences, both within and between groups. The following criteria were employed for interpreting the magnitude of the ES: trivial (<0.2), small (0.2–0.6), moderate (0.6–1.0) and large (>1.0). All calculations were performed using JASP 0.9.2 for Mac (University of Amsterdam, Netherlands). The level of significance was set as p< 0.05.

## Results

### Intrinsic motivation

Participants in the EXP group showed a significant, moderate improvement in the intrinsic motivation to training (p< 0.05, ES = 1.28, 95% CI = 0.30, 2.22), while participants in the CON group showed non-significant decreases in this variable (p > 0.05, ES = -0.75, 95% CI = -1.55, 0.12). A moderate, significant between-group difference was observed for this variable (p < 0.05, ES = 0.58). No group showed significant post-study changes in the demotivation scale (p > 0.05).

### Muscle thickness

**Fig 1:**
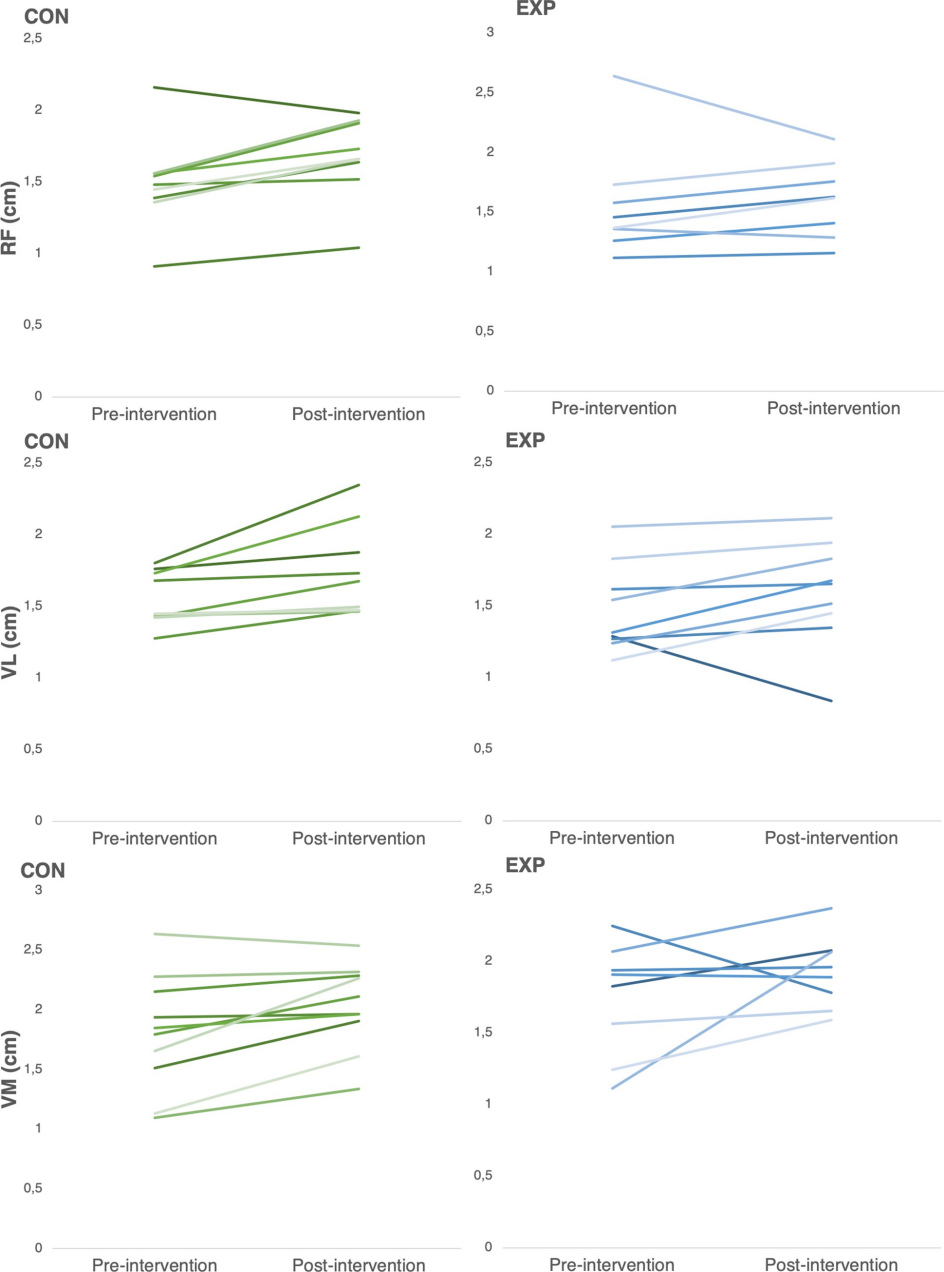
Pre and post intervention scores in muscle thickness for the EXP and CON groups.

No significant differences were observed between groups in any of the MT variables analyzed at pre-intervention (p> 0.05). Also, normality of the distributions and homogeneity of the variances were confirmed for both groups at pre-intervention in those MT variables. Fig 1 illustrates changes in MT for the individual quadriceps’ muscles. Both the EXP and CON group showed large, statistically significant increases in MT of the VL (EXP: p <0,05, ES = 1.43, 95%CI = 0.4, 2.42; CON: p <0,05, ES = 1.03, 95%CI = 0.19, 1.83). Trivial, non-statistically significant differences were observed between groups (p > 0.05, ES<0.2).

MT of the RF increased significantly in both EXP and CON (EXP: p<0,05, ES = 1.19, 95%CI = 0.17, 2.16; CON: p<0,05, ES = 1.05, 95%CI = 0.20, 1.86). No significant between-group differences were noted for change in this outcome (p > 0.05, ES = 0.30).

MT of the VI showed significant increases only in CON; (p <0,05, ES = 1.07, 95%CI = 0.30, 1.80); EXP showed absolute increases in this variable, but results were not statistically significant (p > 0.05, ES = 0.78, 95%CI = 0.17, 1.68). No significant between-group differences were noted for change in this outcome (p > 0.05, ES = 0.27).

### Body composition

Pre- and post-training values of percentage body fat and BMI are shown in Table 2. No significant differences in any measurement were noted from baseline to post study (p >0.05). No significant difference was observed between groups (p >0.05). The ES difference was trivial for BMI (ES<0.2), and small favoring CON for percentage body fat (ES = -0.32).

**Table 2:** Pre-post comparison between the experimental and control group in hypertrophy, anthropometrics, strength and motivation.

	Experimental			Control					
	Pre	Post	Mean Absolute change	Pre	Post	Mean Absolute change	Mean absolute difference between groups (95%CI)	Cohen’s d	p
*Ultrasound imaging*									
VL (cm)	1.54 ± 0.31	1.69 ± 0.26	0.12 ± 0.24	1.57 ± 0.26	1.72 ± 0.35	0.18 ± 0.18	0.05 (-0.12, 0.21)	0.162	0.535
RF (cm)	1.54 ± 0.42	1.62 ± 0.31	0.05 ± 0.25	1.49 ± 0.30	1.67 ± 0.28	0.18 ± 0.17	0.09 (-0.08, 0.26)	0.306	0.275
VI (cm)	1.76 ± 0.43	1.90 ± 0.26	0.15 ± 0.40	1.84 ± 0.44	2.03 ± 0.33	0.22 ± 0.21	0.04 (-0.17, 0.26)	0.276	0.653
*Anthropometry*									
BMI (kg/m^2^)	24.1 ± 2.6	23.9 ± 2.4	0.34 ± 0.49	24.7 ± 2.3	24.6 ± 2.5	0.44 ± 0.96	0.03 (-0.6, 0.7)	0.012	0.931
Body Fat (%)	13.9 ± 2.4	13.9 ± 2.6	0.47 ± 0.98	13.7 ± 2.7	12.7 ± 2.7	-0.4 ± 0.85	-0.8 (-1.8, 0.1)	-0.329	0.062
*Strength*									
Back squat 1-RM (kg)	120.0 ± 25.4	125.3 ± 23.7	10.2 ± 7.3	127.3 ± 37.7	135.5 ± 28.6	11.9 ± 6.8	1.6 (-5.5, 8.8)	0.063	0.636
Bench-press 1-RM (kg)	90.8 ± 20.9	91.5 ±21.8	4.0 ± 4.7	97.2 ± 17.8	101.8 ± 18.9	7.1 ± 4.2	2.5 (-1.7, 6.9)	0.125	0.229
*Motivation scale*									
Intrinsic motivation	4.6 ± 1.3	5.1 ± 1.6	0.5 ± 0.3	5.3 ± 0.8	5.1 ± 1.0	-0.1 ± 0.2	-0.8 (-1.0, -0.5)	-0.580	< 0.001
Demotivation	3.1 ± 0.7	3.5 ± 1.2	0.6 ± 0.7	2.7 ± 1.1	3.2 ± 1.2	0.4 ± 0.9	-0.1 (-1.2, 0.9)	-0.112	0.779

VL: vastus lateralis; RF: rectus femoris; VI: vastus intermedius

### Maximal strength

Both the EXP and CON group showed large, statistically significant increases in the bench press (EXP: p <0.05, ES = 0.84, 95%CI = 0.09, 1.55; CON: p <0.05, ES = 1.68, 95% CI = 0.66, 2.7) and back-squat (EXP: p <0.05, ES = 1.40, 95% CI = 0.49, 2.27; CON: p <0.05, ES = 1.75, 95% CI = 0.66, 2.78). Trivial, non-statistically significant differences were observed between groups (p > 0.05, ES < 0.2). See Table 2 for details.

## Discussion

The main goal of this study was to investigate the impact of random exercise selection and range of repetitions on MT, body composition, strength and intrinsic motivation. We hypothesized that random selection using a mobile app (AceWorkout) would enhance motivation levels without compromising improvements in study outcomes. Our hypothesis was confirmed, since the EXP group showed higher levels of motivation throughout the course of the training program and muscular outcomes were generally similar between conditions.

We attempted to isolate the effect of exercise selection by controlling for other RT variables. Both groups performed a total of 18 sets per muscle group per session for the lower body and 9 sets per session per movement pattern (pushing and pulling) for the upper body. Moreover, all participants trained in a range of 6 and 12 repetitions with total training volume equated and each set carried out to volitional muscle failure. These controls allowed us to more confidently draw causality as to how exercise selection impacted the studied outcomes.

A novel finding of our study was that only the EXP group significantly increased motivation levels from pre- to post-study; motivation levels in CON slightly declined. These findings suggest that varying exercise selection may be an important component for enhancing motivation to perform RT. This is an important finding, as evidence indicates that motivation is linked to exercise adherence in different populations [18,19]. Thus, developing strategies that increase motivation to resistance training might help in achieving long-term improvements in fitness and health and reduce the high drop-out ratio observed in fitness centers, that can be up to 80% after 24 weeks of training in some populations [19]. For example, it has been shown that increasing the levels of motivation to resistance training can significantly increase physical, psychological and social parameters in the elderly [20].

Research investigating the effects of exercise selection on muscle hypertrophy is scarce. The most pertinent study on the topic was carried out by Rauch et al. [12], who compared performing a predetermined list of exercises to self-selecting exercises based on individual preferences. Findings showed no between-group differences in LBM (as measured by DXA), although only the group that self-selected exercises showed significant increases from pre- to post-study. These results somewhat deviate from those in our study. The reasons for the discrepancy may be related to the fact that Rauch allowed subjects to choose exercises in an auto-regulated fashion whereas we randomly rotated exercises in the EXP group. Freedom of choice may allow subjects to select exercises that are more suited to their body type and liking, perhaps providing a greater stimulus for adaptation. It also should be noted that we employed more accurate site-specific measures of muscle growth (ultrasound) versus their use of DXA, which may help to further explain inconsistencies between findings.

In another study on the topic, Fonseca et al [11] reported greater increases in muscle cross sectional of the rectus femoris and vastus medialis (obtained by magnetic resonance imaging) when performing a variety of lower body exercises over the course of the study period compared to just the squat. Although these findings are intriguing and suggest a benefit to varying exercise selection, the study differed from ours in several ways. For one, the non-varied groups in Fonseca et al (2014) performed only a single exercise (squat) while our study involved multiple exercises for both conditions. Moreover, exercise variation in our study was random while Fonseca et al (2014) maintained a set schedule. Finally, our subjects were well-trained whereas theirs were untrained. Thus, it is difficult to compare and contrast findings between the two studies.

In regard to strength gains, both conditions showed significant pre- to post-study improvements in all three measurements with no statistical differences observed between groups; ES values were trivial in all of the studied strength-related outcomes. However, gross changes reveal a greater improvement in CON versus EXP for the 1RM bench press (4.7% VS 0.77%, respectively). This could be attributed to motor learning effects, since CON performed the bench press every session whereas EXP performed it with a lower frequency due to the random exercise prescription.

Our study had several limitations that should be acknowledged. For one, MT measurements were performed only on the quadriceps muscles; we therefore cannot generalize results to the upper body muscle groups. Moreover, we obtained MT measurements at a single site along each quadriceps head. There is evidence that the quadriceps hypertrophies in a regional manner [21]. Thus, it is possible that more proximal and/or distal sites may have experienced differential hypertrophy from the imposed alterations in exercise selection. In addition, while the sample was comprised of trained men (at least 2 years of RT experience), the particulars (consistency, technique, effort, etc) of their training approach varied from subject to subject. This may increase standard deviation, thereby reducing statistical power and increasing the possibility of a type II error. In addition, although we attempted to control resistance training variables (e.g. repetition volume, intensity of load, rest interval), other variables such as total volume load per muscle group, volume progression, and muscle activation couldn’t be controlled. These are inherent limitations that will arise when randomizing different exercises, therefore clouding the ability to determine whether results are attributed to varying exercise selection versus other confounding factors specific to the given routines. Finally, the relatively short intervention duration (eight weeks) may not have allowed sufficient time to realize meaningful differences in trained subjects.

## Conclusions

Our study showed that randomization of exercise selection in trained men may enhance intrinsic motivation to exercise over an 8-week RT program. These results were obtained with relatively similar changes in muscular adaptations, although some outcomes appeared to be slightly attenuated from frequent rotation of exercises. The findings indicate that regularly changing exercise selection could help to enhance adherence to RT in those who lack motivation to train.

### Practical applications

There may be a trade-off whereby too frequent rotation of exercises somewhat compromises muscle growth and strength; thus, those who wish to maximize these outcomes may wish to limit exercise variety. A possible solution is to keep more complex, free weight exercises (e.g. squats, deadlifts, rows, etc) in a regular rotation throughout a training cycle and vary movements that have limited degrees of freedom and thus do not require a high degree of motor learning (e.g. leg extensions, machine press, arm curls, etc). Finally and importantly, exercise rotation was carried out randomly, without attention to individual needs and abilities. It is possible that individualized programming whereby exercise selection is carefully manipulated to take into account biomechanical, physiological and anthropometric factors may further enhance muscular adaptations.
